# Supplementary material for: Factors associated with wasting among pediatric cancer patients aged 2–17 years at Uganda cancer institute: A cross-sectional study
Source: PLoS One. 2025 Sep 26;20(9):e0333076. doi: 10.1371/journal.pone.0333076 (PMC12469180; doi:10.1371/journal.pone.0333076)
Supplement: Attachment — Submitted filename: Manuscript review PONE 2025.docx [file pone.0333076.s002.docx]

**Reviewer: Nicolette Nabukeera Barungi**

**Comments**

This is a good study and very relevant. However, there should be revisions as below:

The authors need to be clear about reference 6 which was a study done in Uganda with higher numbers, the authors need to state the setting and time when that study was done so as to justify why they did a similar study. What gaps did they find in this study?

Line 93-96, Authors need to be more clear on the gaps of using weight to determine nutritional status. They should phrase the objective better, indicating that in order to overcome the errors introduced by using weight, they determined nutritional status. Before this, they need a line or 2 on what MUAC assesses with references, its accuracy compared to Weight for age etc.

MUAC has many issues when used for children aged 5 years and above. It is quite unreliable and cut offs were developed in refugee settings and its reliability is also problematic. The authors need to discuss this. It would be good to categorize the ages, below 5 and above 5 years. Almost 80% were above 5 years in your study so this is very concerning. How many had solid tumors?

We need to know the numbers of children at UCI like monthly or annually. It is more informative than the daily attendances and inpatients at any one point. How many inpatients per yearor per month?

Does the unit have general doctors and general paediatricians? Role of nutritionist? Is there routine assessment and education or supplementation? Focus the study setting to what affects the study results and helping with generalizability.

Why child-caregiver pairs and not just the child? What if the child had multiple care takers?

Line 129 has ref 10 yet earlier that study was ref 6. Are you using manual referencing? The two are the same reference.

Line 134, “individuals who did not assent”? How about those who did not consent?

Line 148: MUAC was the study outcome but it has not been described in the methods. Which MUAC tapes were used for who? (Seen part of it in line 160-165). It needs to be in one place, not scattered in different places.

IIPAN is not described in full in the manuscript. What is it and why should it be used and not just the WHO?

Line 150: I thought your inclusion criteria was confirmed cancer. If so, then you would not need to exclude suspects because they do not qualify.

Mramba’s study s ref 23 not 22

It is not clear at what point you enrolled the children. How did you handle the admitted children especially asking about the 24 hour recall when they have been on the ward for a while?

Table 1; caregiver employment- does this cover self employment? What does it mean? Father or mother? Or both?

Table 1 or 2 should indicate how many are outpatients and inpatients and they need to be also analyzed as variables affecting nutritional status.

Revise and re-align the references
