# Supplementary material for: Factors associated with wasting among pediatric cancer patients aged 2–17 years at Uganda cancer institute: A cross-sectional study
Source: PLoS One. 2025 Sep 26;20(9):e0333076. doi: 10.1371/journal.pone.0333076 (PMC12469180; doi:10.1371/journal.pone.0333076)
Supplement: Attachment — Submitted filename: Response to Reviewers.docx [file pone.0333076.s005.docx]

26^th^ August, 2025

Dear Editor,

**RE: Response to comments on manuscript titled “*FACTORS ASSOCIATED WITH WASTING AMONG PEDIATRIC CANCER PATIENTS AGED 2-17 YEARS AT UGANDA CANCER INSTITUTE: A CROSS-SECTIONAL STUDY*”**

Thank you for handling the review of our manuscript. We are also grateful to the reviewers who have provided constructive comments that have improved our manuscript. As requested, please see enclosed a point-by-point response for each of the comments provided and a revised version of the manuscript for your further consideration.

**Comment**

**In your method section please include the statement "The parents/ guardians were given a consent form which they signed as an indicator of willingness to participate in the study." in the methods section.**

The methods section has been adjusted to include “The parents/ guardians were given a consent form which they signed as an indicator of willingness to participate in the study” (pages 6-7, Lines 107-109)

**Academic Editor**

**Current comment from the academic Editor: The comment about the sample size calculation and the 70% attainment is a main limitation of the study. Is it possible to request for an amendment to justify the number of children enrolled to the study?**

We thank the reviewer for this important observation. While the calculated sample size was 384 participants, only 270 caregiver-child pairs were enrolled in the study. This is because there was a limited number of eligible pediatric cancer patients attending the Uganda cancer Institute during the four-month data collection period, as well as the exclusion of participants with incomplete or missing records. We have now amended the “methods” and “study strengths and limitations” sections to justify the final sample size achieved and its implications.

**Please note that the attached comments were the previous comments from the second reviewer. Please below;**

**Reviewer 2	The authors need to be clear about reference 6 which was a study done in Uganda with higher numbers, the authors need to state the setting and time when that study was done so as to justify why they did a similar study. What gaps did they find in this study?**

We appreciate your feedback.

Reference 6, conducted by Jeanine et al. (2021), was a retrospective study carried out at the Children’s Cancer Unit of Mbarara Regional Referral Hospital in Uganda. The study analyzed medical records of pediatric cancer patients admitted between May 2017 and 2019, focusing on demographic characteristics, anthropometric measurements at admission, and cancer diagnoses. The study population included children under 16 years of age, and nutritional status was assessed using weight-for-length/height for those under five years, and Body Mass Index-for-age (BMI-for-age) for those aged five years and above.

While this study provided valuable insights, several limitations justify the need for a similar study at the Uganda Cancer Institute (UCI)—the national center of excellence for cancer care. First, the setting of the previous study was a regional referral hospital, whereas UCI is a national referral hospital serving a broader and more diverse population, including more complex and advanced cancer cases. Second, the age range in the study by Jeanine et al. was limited to children below 16 years, therefore excluding older pediatric patients aged 16–17 years, who are also a critical part of the pediatric cancer population. Third, the use of weight-based anthropometric indicators is a limitation, as these measures can be affected by the presence of solid tumors and organomegaly, potentially leading to misclassification of nutritional status.

These methodological and contextual gaps highlight the need for the current study, which aims to provide updated, center-specific data on the prevalence of wasting and its associated factors among pediatric cancer patients aged 2–17 years at UCI, using more appropriate nutritional assessment tools such as the Mid-Upper Arm Circumference (MUAC).

**Line 93-96, Authors need to be more clear on the gaps of using weight to determine nutritional status. They should phrase the objective better, indicating that in order to overcome the errors introduced by using weight, they determined nutritional status. Before this, they need a line or 2 on what MUAC assesses with references, its accuracy compared to Weight for age etc.**

Thank you for your feedback.

We have clarified on the gaps of using weight to determine nutritional status. (Pages 4-5 and Lines 83-102)

We have rephrased the objective better to indicate that MUAC was used to determine nutrition status in order to overcome the errors introduced by using weight. (Page 5 and lines 96-102)

Information on MUAC assessment, reference values and accuracy compared to weight for age and BMI for age has been added age [Sisay et al 2020](https://pubmed.ncbi.nlm.nih.gov/33121554/). (Page 5 and lines 88-102)

**Reference**

Sisay BG, Haile D, Hassen HY, Gebreyesus SH. Mid-upper arm circumference as a screening tool for identifying adolescents with thinness. Public Health Nutr. 2021 Feb;24(3):457-466. doi: 10.1017/S1368980020003869. Epub 2020 Oct 30. PMID: 33121554; PMCID: PMC10195468.

**MUAC has many issues when used for children aged 5 years and above. It is quite unreliable and cut offs were developed in refugee settings and its reliability is also problematic. The authors need to discuss this. It would be good to categorize the ages, below 5 and above 5 years. Almost 80% were above 5 years in your study so this is very concerning. How many had solid tumors?**

Thank you for the review.

Several studies including WHO ([here](https://applications.emro.who.int/emhj/0804_5/emhj_2002_8_4_5_612_618.pdf), [here](https://iris.who.int/bitstream/handle/10665/44129/9789241598163_eng.pdf)), [Sisay et al 2020](https://pubmed.ncbi.nlm.nih.gov/33121554/) and [Mramba et al 2017](https://pubmed.ncbi.nlm.nih.gov/28774873/), indicate that MUAC is comparably as accurate as BMI for age Z-scores among children and adolescents.

Categorization of age into below 5 and above 5 years was done in tables 1 and 3.

(Pages 11-13, 15-17 and Lines 222-223, 270-271 respectively)

According to our study, 73.3 % of the pediatric cancer population had solid tumors as shown in table 2. (Page 13 and Line 231)

**Reference**

Sisay BG, Haile D, Hassen HY, Gebreyesus SH. Mid-upper arm circumference as a screening tool for identifying adolescents with thinness. Public Health Nutr. 2021 Feb;24(3):457-466. doi: 10.1017/S1368980020003869. Epub 2020 Oct 30. PMID: 33121554; PMCID: PMC10195468.

Mramba L, Ngari M, Mwangome M, Muchai L, Bauni E, Walker AS, Gibb DM, Fegan G, Berkley JA. A growth reference for mid upper arm circumference for age among school age children and adolescents, and validation for mortality: growth curve construction and longitudinal cohort study. BMJ. 2017 Aug 3;358:j3423. doi: 10.1136/bmj.j3423. PMID: 28774873; PMCID: PMC5541507.

**We need to know the numbers of children at UCI like monthly or annually. It is more informative than the daily attendances and inpatients at any one point. How many inpatients per year or per month?**

Thank you for the review.

Annually, Uganda reports about 3000 new pediatric cancer cases, however, only 30% receive care at cancer centers as shown [here](https://www.afro.who.int/countries/uganda/news/who-supports-development-child-and-adolescent-cancer-control-strategy-uganda). UCI has an inpatient capacity of 40 beds. (Page 4 and Lines 76-78)

**Reference**

<https://www.afro.who.int/countries/uganda/news/who-supports-development-child-and-adolescent-cancer-control-strategy-uganda>

**Does the unit have general doctors and general pediatricians? Role of nutritionist? Is there routine assessment and education or supplementation? Focus the study setting to what affects the study results and helping with generalizability.**

We appreciate your feedback.

Yes, the unit has general doctors, general pediatricians, oncology pediatricians and a nutritionist.

However, the assessment, education and supplementation are not routine due to the high nutritionist: patient ratio. This is because the nutritionist is solely responsible for assessment, education and counselling, supplementation, meal preparation for about 150 children per day hampering establishment of a routine.

In order to align our study to what affects the study results and helping with generalizability, our study setting has been focused to include that currently UCI serves as the East African Centre of excellence in oncology to help with generalizability of the study findings.

Furthermore, the lines 119-122, have captured that the “Pediatric Unit has a team of qualified pediatric oncologists, nurses, a nutritionist, and other healthcare professionals who collaborate to serve approximately, 150 patients daily (in- and outpatients)”. This information highlights the presence of one nutritionist serving 150 pediatric daily which affects the implementation of routine assessment, education and integration of nutrition care into cancer management. (Page 6 and Lines 119-122)

**Why child-caregiver pairs and not just the child? What if the child had multiple care takers?**

Thank you for your feedback.

Our study considered child caregiver pairs and not just children because parents or caregivers are considered sufficient to accurately to represent their children. Supporting evidence for validity of this choice is available here [(Wallace et al 2018).](https://pmc.ncbi.nlm.nih.gov/articles/PMC6115856/)

Furthermore, a proportion of pediatric cancer patients were minors and therefore not fully capable of independently responding to study questions. The physiological status/ wellbeing of some of the pediatric cancer patients also affected their ability to respond to the questions themselves, necessitating the inclusion of caregivers.

**Reference**

Wallace A, Kirkpatrick SI, Darlington G, Haines J. Accuracy of Parental Reporting of Preschoolers' Dietary Intake Using an Online Self-Administered 24-h Recall. Nutrients. 2018 Jul 29;10(8):987. doi: 10.3390/nu10080987. PMID: 30060605; PMCID: PMC6115856.

**Line 129 has ref 10 yet earlier that study was ref 6. Are you using manual referencing? The two are the same reference.**

Thank you for the review.

The two references are the same, and the duplicate has been deleted. (Page 23 and Lines 385-387, 399-402)

**Line 134, “individuals who did not assent”? How about those who did not consent?**

Thank you for the review.

The statement “individuals who did not assent” has been replaced with “individuals who did not consent”. (Page 7 and Line 142)

**Line 148: MUAC was the study outcome but it has not been described in the methods. Which MUAC tapes were used for who? (Seen part of it in line 160-165). It needs to be in one place, not scattered in different places.**

Thank you for your feedback.

MUAC was the tool of measurement for the outcome variable (wasting). This has been clarified in the method section.

The MUAC tapes used were color coded, flexible, non-tearable and non-stretchable. Tapes of measuring ranges of 26.5cm, 40.5cm, and 45.5cm were used for children 6-59 months and 6-10 years, adolescents 10-15 years and 15-18 years respectively. The MUAC tapes were standard (S0145620 MUAC, Child 11.5 Red/ PAC- 50). (Pages 8-9 and Lines 165-170)

**The information on MUAC has been placed in the methods section.**

**IIPAN is not described in full in the manuscript. What is it and why should it be used and not just the WHO?**

Thank you for your feedback.

IIPAN has been described in the manuscript as the International Initiative for Pediatrics and Nutrition.

Headquartered at Columbia University Irving Medical Center in New York City, IIPAN plays a critical role in nutritional training, research, and advocacy for improved quality of life and survival rates of children with cancer.

In Uganda, IIPAN has pioneered the establishment of the nutrition unit at the pediatric cancer ward at the Uganda Cancer Institute.

**Line 150: I thought your inclusion criteria was confirmed cancer. If so, then you would not need to exclude suspects because they do not qualify.**

Thank you for your feedback.

The exclusion of suspects has been eliminated. (Page 8 and Line 157)

**Mramba’s study s ref 23 not 22**

We appreciate your feedback.

This has been rectified. Mramba’s study is now ref 20. (Page 25 and Lines 426-429)

**It is not clear at what point you enrolled the children. How did you handle the admitted children especially asking about the 24 hour recall when they have been on the ward for a while?**

Thank you for your feedback.

Children were enrolled, on basis of being a pediatric cancer patient on either inpatient or outpatient ward.

Children admitted on the ward were still asked about the 24-hour dietary intake despite being on the ward for a while. This is because children are still allowed to eat whether on ward or at home.

**Table 1; caregiver employment- does this cover self-employment? What does it mean? Father or mother? Or both?**

Thank you for the review.

Yes, caregiver employment does cover self-employment.

A caregiver is any person who is directly taking care of the pediatric cancer patient aged 2-17 years regardless of his/ her relationship with the child. A caregiver may be the child’s father or mother.

**Table 1 or 2 should indicate how many are outpatients and inpatients and they need to be also analyzed as variables affecting nutritional status.**

Thank you for your feedback.

Table 2 and 3 now indicate how many are outpatients and inpatients were and the analysis of how these variables affect nutritional status has been done. However, no statistically significant association was found between the type of ward the patient was and the prevalence of wasting. (Pages 12-13,15-17 and Lines 231, 270-271 respectively)

**Revise and re-align the references**

We appreciate your feedback.

This has been rectified

Sincerely,

Daisy Wannyana, on behalf of the co-authors.
